# Tobacco use and associated factors among adults in Ethiopia: further analysis of the 2011 Ethiopian Demographic and Health Survey

**DOI:** 10.1186/s12889-015-1820-4

**Published:** 2015-05-13

**Authors:** Yihunie Lakew, Demewoz Haile

**Affiliations:** Ethiopian Public Health Association, Addis Ababa, Ethiopia; Department of Public Health, Madawalabu University, College of Medicine and Health Sciences, Bale-Goba, Ethiopia

## Abstract

**Background:**

Tobacco is one of the leading preventable causes of non-communicable diseases. Previous studies gave due emphasis only for cigarette smoking with little attention given for other types of tobacco use. This study describes the prevalence of all common forms of tobacco use and identify associated factors among adults in Ethiopia.

**Methods:**

The study used data from the 2011 Ethiopian demographic and health survey. An index was constructed from yes or no responses for common types of tobacco use. Bivariate and multivariate logistic regression statistical models were employed to determine associated factors with tobacco using adjusted odds ratios (AOR) and their 95 % confidence intervals (CI).

**Results:**

The overall prevalence of tobacco use was 4.1 % [95 % CI: (3.93–4.37)]. The highest prevalence 16.9 % [95 % CI: (11.02–23.76)] in Gambella and the lowest 0.8 % [95 % CI: (0.48–1.29)] in Tigray regions were reported. The odds of tobacco use in the age group 20–24 and 45–49 years were [AOR = 2.3; 95 % CI: (1.60–3.21)] and [AOR = 9.1; 95 % CI: (6.06–13.54)] more likely to use tobacco, respectively, as compared to the age group 15–19 years. Traditional religion [AOR = 5.5; 95 % CI: (3.96–7.55)], Catholics [AOR = 3.40; 95 % CI: (2.03–5.69)] and Islamic followers [AOR = 2.8; 95 % CI: (2.31–3.32)] had higher odds of using tobacco as compared to Orthodox religion followers. Adults in the poorest wealth quintile were [AOR = 1.4; 95 % CI: (1.05–1.79)] more likely to use tobacco as compared to the richest wealth quintile. The odds of tobacco use among males were higher as compared to females [AOR = 13.08; 95 % CI: (10.24–16.72)]. Formerly married adults were [AOR = 1.71; 95 % CI: (1.20–2.34)] more likely to use tobacco as compared to never married. Adults who were professionally working [AOR = 0.49; 95 % CI: (0.29–0.85)] had less likely to use tobacco as compared to non-working adults. However, adults who were working in sales, skilled and unskilled occupations had [AOR = 1.6; 95 % CI: (1.18–2.24)], [AOR = 1.7, 95 % CI: (1.21–2.50)] and [AOR = 3.8 95 % CI: (2.27–6.23)] more likely to use tobacco, respectively, as compared to non-working adults. Individuals who had experience of child death were [AOR = 1.4; 95 % CI: (1.17–1.63)] more likely to use tobacco as compared to their counterparts.

**Conclusion:**

The overall prevalence of tobacco use seems low in Ethiopia. However, a significant regional variation of tobacco use was observed. A tailored public health interventions targeting regions with high prevalence of tobacco use is recommended.

## Background

Tobacco is a highly addictive substance that causes strong physiological responses in the human body [1]. It has been recognized as a causes of human suffering and socio-economic problems [2]. Tobacco use is one of the leading causes of preventable deaths in the world [3-6]. Presently, it has become a significant public health concern worldwide [2, 7]. Direct tobacco use is estimated to cause five million deaths a year globally, with indirect exposure leading to an additional 600,000 deaths [1]. A global adult tobacco survey conducted in three billion individuals from 16 countries showed that about 48 · 6 % [with 95 % confidence interval (CI): 47 · 6–49 · 6)] of men and 11 · 3 % [95 % CI: 10 · 7–12 · 0)] of women were tobacco users [8]. A recent estimate revealed that, about 400 million adults worldwide will be killed by smoking alone between 2010 and 2050. Most of the deaths will occur in the age group 30–69 years, losing decades of productive life [9].

The economic costs of tobacco use are equally devastating. Besides the direct costs of treating tobacco-related diseases, economic productivity is lost due to preventable illness and premature deaths among users. Unnecessary expenditures to purchase tobacco also contribute to household poverty, malnutrition and illiteracy rates particularly within resource limited settings [10]. The total burden caused by tobacco products more outweighs than any economic benefit from their manufacture and sales [11]. In the United States of America alone more than 289 billion US dollar lost yearly due to tobacco smoking [12]. Even though, there is a lack of country-specific research on the economic costs of tobacco use in low-and middle-income countries, an increasingly growing tobacco consumption has been observed in Africa [13]. Currently, tobacco use in Africa is increasing as the tobacco industry shifts its marketing focus from the West to “areas of strong growth” in Africa and Asia [14].

One important public health approach for controlling tobacco use is to design and implement appropriate policy. The WHO Framework Convention on Tobacco Control (WHO FCTC) was designed as a global response to tobacco epidemics [15]. However, most African countries have thus far failed to achieve their obligations outlined in the WHO FCTC [7]. The implementing guidelines for WHO FCTC includes prohibition of promotion of electronic and print media of tobacco, demoralized and regulate “socially responsible” activities carried out by the tobacco industry such as sponsorship accepted for sport events and any other small grants [16]. Ethiopia is one of the countries in sub-Saharan Africa shares the burden of tobacco epidemics. The country is ranked at 66^th^ in producing tobacco and contributing about 0.1 % to the world led by China, Brasil and India [17]. The Ethiopian parliament has ratified the international tobacco control convention (WHO FCTC) on 25 March 2014 [16]. This is an important step for reducing tobacco use in the country. However, there is paucity of information analyzed at national and regional levels to effectively implement the WHO FCTC guideline. Most previous studies gave due emphasis only for cigarette smoking with very little or no attention given for other types of tobacco use [18, 19]. In this regard understanding all common forms of tobacco and its associated factors is also an important step for targeting interventions. Therefore, this study is intended to determine levels of all common types of tobacco use and identify factors associated with tobacco use among adults in Ethiopia.

## Methods

### Data type and study design

This study used secondary data from the 2011 Ethiopian demographic and health survey (EDHS). The survey followed an international DHS methodology and is conducted at five years interval. The EDHS was designed to provide population and health indicators at national (urban and rural) and regional levels. The 2011 EDHS samples were selected using a stratified, two-stage cluster sampling design. The sample included 624 enumeration areas, of which 187 were in urban areas and 437 in rural areas. Representative samples of 16,702 households from 11 administrative regions were included during interview, of which 11,590 were from rural settings. A total of 30,625 adults aged between 15 and 59 years were interviewed and of which 16,515 accounted for women respondents. Like many other countries, no defined male to female ratio per household was interviewed in the 2011 EDHS. Males were interviewed in an alternative households. The detailed methodology is found elsewhere [20, 21].

### Data extraction

The 2011 EDHS data were downloaded from the Measure DHS website (http://www.dhsprogram.com) in SPSS format with permission. After understanding the detailed data coding, further data recoding was completed. Based on published literature, socio-demographic and economic variables, exposure to mass media and tobacco use indicators were extracted from EDHS 2011 men and women datasets. The two datasets were merged for the analysis.

### Measurement of variables

In our analysis the dependent variable was tobacco use. We defined “tobacco use” if respondents used any form or type of tobacco listed in the 2011 EDHS data. All common types of tobacco use were assessed by asking respondents about current use of any tobacco type. Cigarette smoking was assessed by asking questions such as, “Do you currently smoke cigarettes?” Other types of tobacco use was assessed by asking the respondents, “What (other) type of tobacco do you currently smoke or use like pipe, chewing tobacco, snuff/suret, shisha, gaya or any other type?”. We found six types of tobacco use in the 2011 EDHS database: cigarette smoking (yes, no), chewing tobacco (yes, no), snuff (yes, no), shisha (yes, no), gaya (yes, no) and any other types of tobacco (yes, no)”. No responses of each tobacco type were recoded as “0” and considered as “did not use any type”, while yes responses were recoded as “1” and considered as respondents used any one of the types. Then, all yes and no answers were added to create an index from any types of tobacco use. A score ranged from 0 to 6 was found and zero score was labeled as “non-tobacco users” and score from 1 to 6 was labeled as “tobacco users”. Finally, a dummy variable containing “0” if the adults had not used any types and “1” if the adults had used one or more tobacco types was created.

Based on literature review [18, 19] and availability of variables in the 2011 EDHS data, wealth index constructed by EDHS from household assets and characteristics, age groups, occupational status, child death experience, categories of religion, residence, educational status, mass media exposure indexed from reading newsletter, listening radio and television, gender, and marital status were selected to be potentially associated with tobacco use.

### Method of statistical analysis

We used “svy” in STATA version 11 to weight the survey data and do the analyses. Sample weights were applied in order to compensate for the unequal probability of selection between the strata that has been geographically defined as well as for non-responses. A detailed explanation of the weighting procedure can be found in the EDHS methodology report [20, 21]. Descriptive statistics were used to show the overall weighted prevalence of tobacco use across regions, gender, place of residence and different forms of tobacco use. Bivariate and multivariate logistic regressions were carried out to determine the factors associated with tobacco use. Variables found statistically significant at *p*-value <0.25 during bivariate analysis were considered for multivariable logistic regression model [22]. This *p*-value cutoff point was used not to remove many variables in the bivariate stage that may have potential effect during multivariate analysis. All tests were two-sided and a *p*-value <0.05 was considered statistically significant during multivariable statistical model. Both crude and adjusted odds ratios were calculated with 95 % confidence interval (CI).

### Ethical consideration

The original DHS data were collected in conformity with international and national ethical guidelines. Ethical clearance for the original survey was provided by the Ethiopian Public Health Institute Review Board, the National Research Ethics Review Committee (NRERC) at the Ministry of Science and Technology, the Institutional Review Board of ICF International and the Centers for Disease Control and Prevention (CDC). The data for this study were downloaded and used after the purpose of the analysis was communicated and approved by the Measure DHS.

## Results

The survey included un-weighted total adult populations of 30,625 in the age range from 15 to 59 years old. Among the respondents, 16,515 (53.9 %) were females. About 21,080 (68.8 %) respondents were from rural areas. With regard to educational status, about 42 % were not educated and 41 % were under the category of primary education. The proportion of Christians (Orthodox, Protestant, and Catholic) was 60.7 % followed by Islam 37.5 %. The mean age of respondents was 29.0 with standard deviation (SD) of 10.5.

One thousand two hundred sixty eight respondents reported that they had used at least one types of tobacco. As indicated in Table 1, the overall prevalence of all forms of tobacco use was 4.1 % [95 % CI: (3.93–4.37)]. The prevalence of tobacco use in men was 8.1 % [95 % CI: (7.67–8.57)] whereas it was 0.8 % [95 % CI: (0.62–0.89) in women. The prevalence of cigarette smoking was 3.1 % [95 % CI: (2.91–3.30)]. The prevalence of chewing tobacco and snuff was 0.7 % [95 % CI: (0.57–0.76)] and 0.4 % [95 % CI: (0.29–0.43)], respectively. As shown in Table 2, the highest prevalence of tobacco use was in Gambella region with 16.9 % [95 % CI: (11.02–23.76)], followed by Harari region with 16.7 % (95 % CI: 9.79–24.92)]. The prevalence of tobacco use was 15.4 % [95 % CI: (10.17–22.87)] in Dire Dawa and 12.6 % [95 % CI: (10.10–15.43)] in Somali regional state. The lowest prevalence of tobacco use was reported in Tigray region 0.8 % [95 % CI: (0.48–1.29)].

**Table 1:** Prevalence of indexed tobacco use with all common forms by gender in Ethiopia, 2011

Tobacco use items	Total number of respondents*	Male	Female	Both Sexes
	*n* = 30,626	weighed prevalence 95 % CI	weighed prevalence 95 % CI	weighed prevalence 95 % CI
Cigarette	30,616	6.5(6.08–6.90)	0.2(0.15–0.29)	3.1(2.91–3.30)
Chew tobacco	30,599	1.3(0.12–1.50)	0.1(0.07–0.17)	0.7(0.57–0.76)
Snuff	30,599	0.7(0.54–0.81)	0.1(0.05–0.14)	0.4(0.29–0.43)
Shisha	30,599	0.2(0.11–0.25)	0.2(0.10–0.22)	0.2(0.12–0.21)
Gaya	30,599	0.3(0.22–0.40)	0.2(0.18–0.33)	0.3(0.21–0.33)
Others	30,599	0.2(0.12–0.27)	0.01(0.002–0.04)	0.1(0.06–0.13)
Index of scores	30,597	8.1(7.67–8.57)	0.8(0.62–0.89)	4.1(3.93–4.37)

*The total number of each type of tobacco use and their index are disproportionate because of multiple question items and missing values. The numbers are weighted using EDHS weighting factor variable

**Table 2:** Current prevalence of tobacco use among the adult population across regions, residence and gender in Ethiopia, 2011

Basic characteristics	Weighted total number of respondents	Weighted prevalence of tobacco use 95 % CI
**Administrative regions**		
Tigray	1,964	0.8(0.48–1.29)
Afar	256	15.7(11.56–20.46)
Amhara	8,334	1.5(1.26–1.78)
Oromiya	11,398	5.3(4.90–5.72)
Somali	596	12.6(10.10–15.43)
Benishangul-gumuz	323	9.4(6.47–12.83)
SNNPR*	5,758	4.4(3.89–4.95)
Gambella	132	16.9(11.02–23.76)
Harari	92	16.7(9.79–24.92)
Addis Ababa	1,620	3.9(3.03–4.92)
Dire Dawa	127	15.4(10.17–22.87)
**Residence**		
Urban	7,032	4.3(3.84–4.79)
Rural	23,565	4.1(3.85–4.36)
**Sex**		
Male	14,101	8.1(7.66–8.56)
Female	16,497	0.8(0.67–0.95)
Total	30,598	4.1(3.93–4.37)

*Southern nation, nationalities and people region

**Fig. 1:**
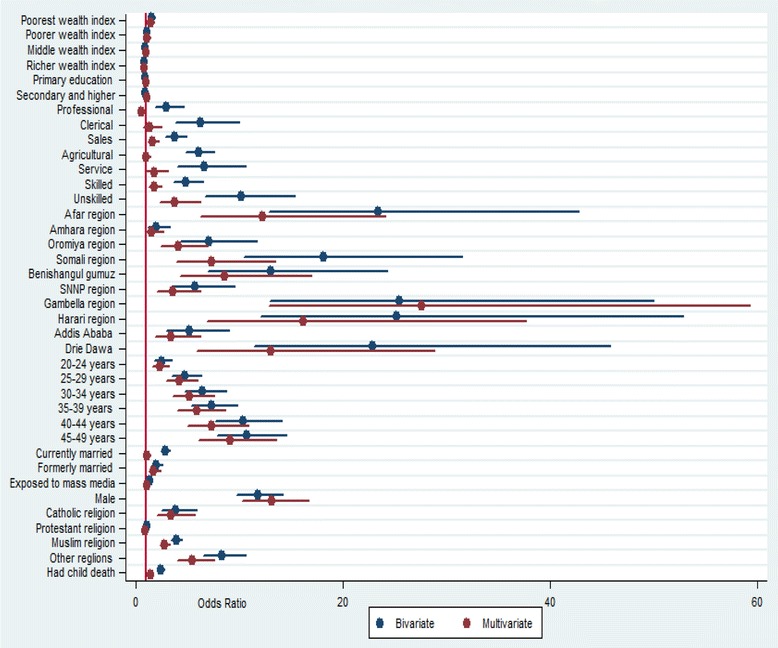
Bivariate and multivariate analysis to indentify factors associated with tobacco use among adult population in Ethiopia, 2011

Place of residence (urban-rural) had no statistically significant association with tobacco use in the bivariate analysis and therefore excluded in the multivariable model. As indicated in Table 3 and Fig. 1, exposure to mass media and educational status were not significantly associated with tobacco use in the multivariable model. Variables including administrative region, wealth index, age, occupation, child death experience, religion, sex and marital status were significantly associated with tobacco use.

**Table 3:** Binary logistic regression analysis to show factors associated with tobacco use among Ethiopian adults, 2011

Variables	Crude OR with 95 % CI	AOR with 95 % CI
**Occupation type**		
Not working	1.00	1.00
Professional	3.0(1.88–4.65)**	0.49(0.29–0.85)*
Clerical	6.2(3.85–10.01)**	1.3(0.73–2.48)
Sales	3.7(2.84–4.91)**	1.6(1.18–2.24)*
Agricultural	6.0(4.78–7.61)**	1.0(0.74–1.37)
Service	6.6(4.03–10.65)**	1.7(0.99–3.09)
Skilled	4.9(3.60–6.54)**	1.7(1.21–2.50)*
Unskilled	10.2(6.72–15.39)**	3.8(2.27–6.23)**
**Administrative regions**		
Tigray	1.00	1.00
Afar	23.4(12.82–42.74)**	12.2(6.22–24.09)**
Amhara	2.0(1.15–3.32)*	1.5(0.88–2.68)
Oromiya	7.0(4.24–11.67)**	4.1(2.36–6.98)**
Somali	18.1 [10.40–31.54]**	7.3(3.93–13.48)**
Benishangul-gumuz	13.0(6.97–24.27)**	8.5(4.28–16.98)**
SNNPR	5.7(3.42–9.58)**	3.6(2.02–6.27)**
Gambella	25.4(12.94–49.98)**	27.6(12.83–59.30)**
Harari	25.2(12.02–52.86)**	16.1(6.89–37.69)**
Addis Ababa	5.1(2.94–8.98)**	3.4(1.84–6.20)**
Dire Dawa	22.9(11.4–45.81)**	13.1(5.91–28.87)**
**Age group**		
15–19	1.00	1.00
20–24	2.5(1.78–3.45)**	2.3(1.60–3.21)**
25–29	4.7(3.48–6.34)**	4.2(1.96–5.98)**
30–34	6.5(4.76–8.77)**	5.2(3.53–7.55)**
35–39	7.3(5.38–9.83)**	5.9(4.04–8.67)**
40–44	10.4(7.66–14.08)**	7.3(4.96–10.90)**
45–49	10.7(7.86–14.53)**	9.1(6.06–13.54)**
**Sex**		
Female	1.00	1.00
Male	11.7(9.74–14.16)**	13.1(10.24–16.72)**
**Marital status**		
Currently not married	1.00	1.00
Currently married	2.8(2.41–3.29)**	1.1(0.85–1.37)
Formerly married	2.0(1.50–2.54)**	1.7(1.20–2.43)*
**Child death experienced**		
No	1.00	1.00
Yes	2.4(2.17–2.73)**	1.4(1.17–1.63)**
**Religion**		
Orthodox	1.00	1.00
Catholic	3.9(2.52–5.89)**	3.4(2.03–5.69)**
Protestant	1.1(0.89–1.33)**	0.86(0.67–1.11)
Muslim	3.9(3.40–4.47)**	2.8(2.31–3.32)**
Others including traditional religion	8.3(6.52–10.65)**	5.5(3.96–7.55)**
**Wealth index**		
Poorer	1.5(1.31–1.81)**	1.4(1.05–1.79)*
Poorest	1.1(0.91–1.29)	1.1(0.82–1.41)
Middle	0.87(0.73–1.04)	0.94(0.72–1.23)
Richer	0.82(0.68–0.98)	0.79(0.62–1.02)
Richest	1.00	1.00

* = *p* < 0.001 ** = *p* < 0.0001

Administrative regions were found as one of the factors significantly associated with tobacco use. Those adults who reside in Afar, Oromiya, Somali, Benshanlgul-gumz, SNNP, Gambella and Harari [AOR = 12.2; 95 % CI: (96.22–24.09), [AOR = 4.1; 95 % CI: (2.36–6.98)], AOR = 7.3; 95 % CI:(3.93–13.48)], [AOR = 8.5; 95 % CI: (4.28–16.98)], [AOR = 3.6; 95 % CI: (2.02–6.27)], [AOR = 27.6; 95 % CI: (12.83–59.30)], and [AOR = 16.1; 95 % CI: (6.89–37.69)] had higher odds, respectively, to use tobacco as compared to Tigray regional state. The odds of tobacco use was also higher in Addis Ababa [AOR = 3.4; 95 % CI: (1.84–6.20)] and Dire Dawa [AOR = 13.1; 95 % CI: (5.91–28.87)] administrative cities as compared to Tigray regional state.

Those adults found in the poorest wealth quintile [AOR = 1.4; 95 % CI: (1.05–1.79)] were more likely to use tobacco as compared to the richest quintile. The odds of tobacco use increased as age increased. Adults in the age groups 20–24 years [AOR = 2.3; 95 % CI: (1.60–3.21)], 25–29 years [AOR = 4.1; 95 % CI: (2.96–5.98)] and 30–34 years [AOR = 5.2; 95 % CI: (3.53–7.55)] were more likely, respectively, to use tobacco as compared to age group 15–19 years. Those adults who found in the age category 40–44 years [AOR = 7.3; 95 % CI: (4.96–10.90)] and 45–49 years [AOR = 9.1; 95 % CI: (6.06–13.54)] had higher odds to use tobacco as compared to age group 15–19 years.

Adults who were professionally working [AOR = 0.49; 95 % CI: (0.29–0.85)] had less likely to use tobacco as compared to non-working adults. However, those adults who were working in sales, skilled and unskilled occupational categories had [AOR = 1.6; 95 % CI: (1.18–2.24)], [AOR = 1.7, 95 %CI: (1.21–2.50)] and [AOR = 3.8 95 % CI: (2.27–6.23)] more likely to use tobacco, respectively, as compared to non-working adults.

Individuals who had experience of child death were more likely to use tobacco as compared to their counterparts [AOR = 1.4; 95 % CI: (1.17–1.63)]. Catholic religion followers [AOR = 3.40; 95 % CI: (2.03–5.69)], traditional religion followers [AOR = 5.5; 95 % CI: (3.96–7.55)] and Islamic religion followers [AOR = 2.8; 95 % CI: (2.31–3.32)] have had higher odds of using tobacco as compared to Orthodox religion followers. The odds of tobacco use among males was higher as compared to females [AOR = 13.08; 95 % CI: (10.24–16.72)]. Formerly married individuals were more likely to use tobacco as compared to never married individuals [AOR = 1.71; 95 % CI: (1.20–2.34)] (Table 3 and Fig. 1). The vertical line in Fig. 1 represents odds ratio of 1. Variables with odds ratio on this reference line have no association with tobacco use. Variables with odds ratio above the reference line have a higher odds of tobacco use whereas variables with odds ratio below the reference line have lower odds of tobacco use.

## Discussion

This study found that the national prevalence of tobacco use was 4.1 % in Ethiopia with 8.1 % for males and 0.8 % for females, which is much lower than a national study from Madagascar (48.9 % in males and 10.3 % in females) [23]. A national study from Nepal reported more than half of the population were tobacco users. The most common type of tobacco consumption in Ethiopia was cigarette smoking, unlike chewing tobacco in Nepal [19]. This could be due to the fact that most of the South Asians are highest smokeless tobacco users in the world [23, 24]. The social acceptance of tobacco consumption is high in Nepal [19]. The prevalence of cigarette smoking was 7.3 % in Ghana [18] which is also higher than our finding. In Ghana, there are Europe based companies producing tobacco [25]. The difference in production and cultivation of tobacco might also contribute to the difference in prevalence of tobacco use between Ghana and Ethiopia. Tobacco use among the Ethiopian adult population is relatively low as compared to most African countries [18, 26]. This low prevalence of tobacco use might be explained by the fact that the production of tobacco in Ethiopia is performed by government-owned enterprises. The national tobacco enterprises do not advertise tobacco use to the general population and smoking is viewed as a bad habit and taboo among Ethiopians [27].

This study revealed variation in tobacco use throughout Ethiopia’s regional states. The highest prevalence was found in Gambella region and eastern Ethiopia (Harari, Dire Dawa and Somali). This variation could be attributed to the availability of contraband cigarettes in those regions [27].

This study identified factors associated with tobacco use. The poorest groups of the population were more likely to use tobacco as compared to the richest quintile group. This finding is consistent with studies from other countries such as Nepal [19], India [28, 29] and Ghana [18]. Martin Boba and his colleagues found that deprivation increased the risk of smoking [30]. Smoking is sometimes considered as self-medication used to regulate moods, manage stress, and to cope up with the strains of material deprivation among the poor [31, 32]. This could be the reason that the poorest are at greater risk for tobacco use as compared to rich individuals. The association of tobacco and poverty forms a vicious circle which is often difficult to escape. The poorest group is more likely to use tobacco and tobacco use increases poverty in many countries [33].

The odds of tobacco use was found to increase among the older age groups. Specifically, individuals who were in an older age group were more likely to use tobacco products as compared to those in the younger age group (15–19 years). This is consistent with a study from Nepal [19], Butajira, Ethiopia [34], Ghana [18], Brasil [35] and Madagascar [23]. This might be due to the fact that older individuals have had a longer time to experience tobacco use and develop tobacco use habits [36]. Individuals who initiated smoking early in life have been found to have less chance of quitting smoking later in life [37]. This could also be due to a lack of appropriate interventions for adults, which recalls the necessity of public health interventions that target this segment of population. Many studies have shown that older age groups were more likely to terminate tobacco use especially smoking as compared to their younger counterparts [38-40].

Our study found that occupation type was associated with tobacco use. Professional working was associated with lower odds of tobacco use as compared to currently non-working adults. The possible justification could be the ethics demanded by professional workers might prevent them from tobacco use. Schools and some offices have internal law which banned tobacco use at their own boundary in Ethiopia. A study in Nepal found that adults in manual occupations had increased odds of using tobacco as compared to professional/clerical service jobs [19]. Occupation type was also significantly associated with tobacco use in Madagascar [23]. This study also found that individuals who had experienced child death had a higher risk of using tobacco as compared to their counterparts. This could be explained by the fact that those individuals who experienced child death may use tobacco to regulate mood, manage stress, and to escape the depression associated with this social problem [41] as a self-medication.

There was a statistically significant difference in tobacco use across different religious groups. Islamic religion followers were more likely to use tobacco as compared to Orthodox religion followers. This finding mirrors a number of studies conducted in Ethiopia [34] and abroad [18, 29, 42]. In Ethiopia, especially in Harari and Somali regional states where tobacco use is more prevalent which might be attributed by the prevalence of contraband cigarette in these regions. On the other hand, in these regions the majority of the population are Islamic religion followers. So that these Islam community reside in these regions is exposed to those contraband cigarette. This could also be the reason why this study found that those Islamic religion followers have had higher odds of using tobacco.

Males had higher odds to use tobacco as compared to females. Similarly, most studies in Africa have shown that tobacco use, especially by smoking, is associated with males [36, 39, 43-45]. In Ethiopia, tobacco use in females is condemned by the community and results in stigma and discrimination. Females are more socially restricted than their male counterparts [36]. This logically reduces the chance of female tobacco use in Ethiopia. The odds of tobacco use among formerly married individuals was 70 % higher as compared to never married individuals. This could be due to the fact that formerly married individuals might use tobacco to relieve their stress or loneliness. On the other hand, divorce could be one of the social consequences of tobacco use. Tobacco use might cause conflict among couples and result in divorce.

This is a secondary data analysis which missed key variables that potentially determine tobacco use in a wider perspective. Potential explanatory variables such as availability, affordability, health risk awareness and knowledge issues in the community were not assessed. The other limitation of this study is a social desirability bias to report tobacco use behaviour; sometimes tobacco use is associated with stigma. This study only attempted to assess the prevalence of current tobacco use at the time of the EDHS. It did not represent the prevalence of ever tobacco users. Some regions had small sample size, which questions the accuracy of prevalence estimates per region, so that it should be interpreted in caution.

Despite these limitations, however, this study has enormous public health implications. In the absence of studies attempted to analyze and quantify all forms of tobacco use, the present study could provide useful information at national level. As the population ages and the burden of non-communicable and chronic diseases increases in Africa, including in Ethiopia, the impact of tobacco on health is expected to increase. The health consequences of tobacco use have economic impacts due to costs for treating those with chronic illnesses that are attributed to tobacco use. Health expenditures at the household level increase and affect essential purchases such as for food and shelter. Tobacco use-related deaths tend to occur during the most productive middle-age years, therefore, impacting the economy of the entire nation [7]. Implementation of the ratified WHO FCTC and community awareness about health and socio-economic effects of tobacco use should be strengthened as a policy and for public health interventions. Awareness about the WHO FCTC, as well as about the health and socio-economic impacts of tobacco use in conjunction with key findings of this study, is important and helpful for effective implementation of the proclamation.

## Conclusion

Although the overall prevalence of tobacco use seems relatively low in Ethiopia, there are some regional states, namely Gambella, Harari, Dire Dawa, Afar and Somali that need special attention because of their higher prevalence of tobacco use. Strong system to control contraband cigarettes might benefit these regions. Administrative regions, poorest wealth quintile, older age groups, occupation type, child death experience, Islam, Catholic and other religion including traditional followers, male respondents, and being formerly married were statistically associated factors for tobacco use. Therefore, these factors should be considered for targeting specific public health interventions to reduce tobacco use in Ethiopia. Regions with high prevalence of tobacco use need special attention for intervention.
